# Wearable Devices in Healthcare Beyond the One-Size-Fits All Paradigm

**DOI:** 10.3390/s25206472

**Published:** 2025-10-20

**Authors:** Elena Giovanna Bignami, Anna Fornaciari, Sara Fedele, Mattia Madeo, Matteo Panizzi, Francesco Marconi, Erika Cerdelli, Valentina Bellini

**Affiliations:** Anesthesiology, Critical Care and Pain Medicine Division, Department of Medicine and Surgery, University of Parma, Viale Gramsci 14, 43126 Parma, Italy; anna.fornaciari@outlook.it (A.F.); sara.fedele@studenti.unipr.it (S.F.); mattia.madeo@studenti.unipr.it (M.M.); matteo.panizzi@unipr.it (M.P.); francesco.marconi@unipr.it (F.M.); erika.cerdelli@ao.pr.it (E.C.); valentina.bellini@unipr.it (V.B.)

**Keywords:** clinical monitoring, context-driven selection, continuous monitoring, ethical implications, multiparametric wearable devices, perioperative care

## Abstract

**Highlights:**

- Are wearable devices suitable for all clinical scenarios?
  Wearable devices are not interchangeable in clinical practice due to differences in sensor configuration, connectivity protocols, and validation contexts.
- Which is the main consequence of these findings?
  Context-driven selection may improve clinical utility, safety, and workflow efficiency.
- Which are the main issues that prevent the clinical integration?
  Interoperability and data integration are critical for successful clinical implementation. Ethical and privacy concerns must be addressed for safe and equitable deployment.

**Abstract:**

Wearable devices (WDs) are increasingly integrated into clinical workflows to enable continuous, non-invasive vital signs monitoring. Combined with Artificial Intelligence (AI), these systems can shift clinical monitoring from being reactive to predictive, allowing for earlier detection of deterioration and more personalized interventions. The value of these technologies lies not in absolute measurements, but in detecting physiological parameters trends relative to each patient’s baseline. Such a trend-based approach enables real-time prediction of deterioration, enhancing patient safety and continuity of care. However, despite their shared multiparametric capabilities, WDs are not interchangeable. This narrative review analyzes nine clinically validated devices, Radius VSM^®^ (Masimo Corporation, Irvine, CA, USA), BioButton^®^ (BioIntelliSense Inc., Redwood City, CA, USA. Distributed by Medtronic), Portrait Mobile^®^ (GE HealthCare, Chicago, IL, USA), VitalPatch^®^ (VitalConnect Inc., San Jose, CA, USA), CardioWatch 287-2^®^ (Corsano Health B.V., The Hague, The Netherlands. Distributed by Medtronic), Cosinuss C-Med Alpha^®^ (Cosinuss Gmb, Munich, Germany), SensiumVitals^®^ (Sensium Healthcare Limited, Abingdon, Oxfordshire, UK), Isansys Lifetouch^®^ (Isansys Lifecare Ltd., Abingdon, Oxfordshire, UK), and CheckPoint Cardio^®^ (CheckPoint R&D LTD., Kazanlak, Bulgaria), highlighting how differences in sensor configurations, battery life, connectivity, and validation contexts influence their suitability across various clinical environments. Rather than establishing a hierarchy of technical superiority, this review emphasizes the importance of context-driven selection, considering care setting, patient profile, infrastructure requirements, and interoperability. Each device demonstrates strengths and limitations depending on patient population and operational demands, ranging from perioperative, post-operative, emergency, or post-Intensive Care Unit (ICU) settings. The findings support a tailored approach to WD implementation, where matching device capabilities to clinical needs is key to maximizing utility, safety, and efficiency.

## 1. Introduction

### 1.1. Clinical Monitoring: From Multi-Parameter Wearable Devices to Predictive and Personalized Clinical Management Systems

**Figure 1:**
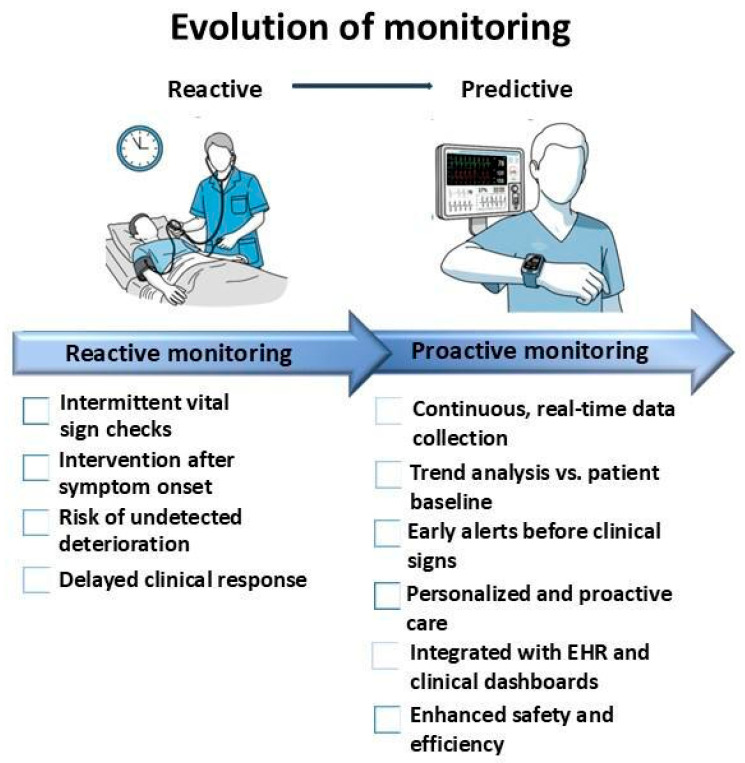
Conceptual illustration contrasting reactive versus predictive clinical monitoring. Reactive management relies on intermittent vital sign measurements and symptom-driven interventions, leaving periods of unmonitored “blind spots.” Predictive management, enabled by wearable devices (WDs), leverages continuous trend analysis to generate early warnings and support timely, personalized interventions.

Clinical monitoring in technologically advanced healthcare systems is undergoing a paradigm shift, from reactive to proactive management (Figure 1), through the integration of wearable devices (WDs). These tools can detect early signs of clinical deterioration through subtle changes in vital parameters, resulting in shorter intervention times, improved clinical outcomes, and reduced length of hospital stay [1]. By generating real-time clinical alerts [2], WDs support timely action. Reactive management simply monitors vital signs at predetermined intervals, triggering interventions after the onset of symptoms, leaving a “care blind spot.” In contrast, proactive/predictive management using WDs and Artificial Intelligence (AI) algorithms aims to monitor physiological trends and generate early warnings, ensuring interventions can be made before clinical deterioration becomes apparent.

The continuous and real-time transmission of physiological data to centralized monitoring platforms and clinical alert systems is possible by connecting WDs to hospital digital infrastructures and telemedicine platforms (Clinical Information Systems, CIS; Electronic Health Record, HER; department and emergency room management systems, etc.).

This integration may assess a “technological ecosystem” capable of transmitting clinical data from sensors to healthcare personnel using wireless data transmission technologies such as Bluetooth Low Energy (BLE) for short-range connections, Wi-Fi for internal hospital coverage, and cellular networks (3G/4G/5G) for home or mobile monitoring.

The integration of digital devices and their data flows can be increased through two models: “edge computing,” in which data is processed directly by the WDs or on an intermediate unit (gateway), even in the absence of a continuous internet connection, and “cloud computing,” in which data is sent to secure remote servers, where algorithms and artificial intelligence models analyze it and return the results to clinical dashboards, accessible in real time. This enables both continuous monitoring and system scalability, centralized algorithm updates, and the possibility of predictive analytics on large amounts of clinical data [3] in order to develop machine learning and deep learning models based on updated, multicenter datasets, thus acquiring greater generalizability because they are exposed to a wider variety of populations, protocols, and clinical contexts [4]. The development of such approaches, including the application of AI, should follow fundamental ethical principles, accountability, safety, autonomy, inclusiveness, transparency, and sustainability, to ensure clinical benefits while limiting inequalities [5]. Algorithm reliability is essential to avoid false positives and decision-making errors, particularly in subacute and perioperative settings, where these systems may represent a strategic asset to enhance intensive and personalized monitoring [6,7,8,9].

### 1.2. Integrating Wearable Devices into Anesthesiological Management

Wearable devices (WDs) facilitate anesthetic risk stratification and functional capacity assessment, even preoperatively, by continuously and non-invasively monitoring physiological parameters such as heart rate (HR), respiratory rate (RR), and oxygen saturation (SpO_2_) [10]. Data from photoplethysmography (PPG) and accelerometers enable objective preoperative risk stratification and functional screening, supporting customized sedation [9,11].

Control of anesthetic depth and analgesia during anesthesia, performed using closed-loop systems, automates the accurate administration of drugs, while nociceptive sensors based on heart rate variability (HRV) and skin impedance provide real-time feedback on tissue damage, reducing opioid use and improving intraoperative stability [12]. Wearable ECGs, combined with machine learning, enable pre-emptive analgesia and remote postoperative pain assessment, optimizing analgesic administration and enhancing recovery [13,14].

### 1.3. From Intensive Care Unit (ICU) to the Ward: The Role of Wearable Devices in Continuous Monitoring

Continuity of clinical monitoring is essential for patient safety, avoiding readmissions to the ICU [15]. Transfer from the ICU to the general ward may expose patients to clinical deterioration that is not detected with intermittent manual monitoring [16]. In the first few hours after surgery, intermittent monitoring is insufficient to promptly detect possible physiological derangement [17], WDs could fill these “care blind spots”, transforming postoperative surveillance [18].

In fact, such monitoring, every 4–6 h in post-operative wards, often becomes insufficient in detecting early conditions, such as hypoxemia and hypotension, underlying clinical deterioration and highlighting the weaknesses of the traditional model [19]. Continuous monitoring represents an early warning opportunity that is still under-exploited [20].

WDs, by performing continuous non-invasive monitoring, bridge the gap between the ICU and ordinary hospital wards by improving patient mobility and the early identification of critical events [21]. This approach supports post-ICU continuity of care and remote monitoring with personalized recovery management [22], and reduces the length of hospital stay in patients undergoing surgery [16]. These devices, combined with predictive algorithms, have shown a high capacity in anticipating critical events up to 14–15 h before clinical manifestation [1]. In addition, the integration of WDs into postoperative early warning score (EWS) systems overcomes the limitations of intermittent monitoring, promoting continuous surveillance based on vital signs analysis. This proactive approach improves the accuracy of early complication identification and supports more timely clinical management [23,24]. An advanced wearable AI system for sepsis (SepAl) has been developed, utilizing vital signs detected by PPG sensors, temperature measurement, and accelerometer data to predict the onset of this condition up to 9.8 h in advance [25]. Similarly, systems such as i-CardiAx have been shown to predict the onset of sepsis with an average advance of 8.2 h [26], and, even in settings such as ICU, have significantly decreased intervention times and clinical outcomes [27].

Thus, data collected by wearable sensors, combined with electronic medical records through deep learning models, allow for a more precise and timely assessment of patient severity, achieving better accuracy than a SOFA (Sequential Organ Failure Assessment) score [28]. Furthermore, neurovegetative parameters such as HRV, temperature, and skin conductance, detected via WDs, have detected early stress, anxiety, and dysautonomia in clinical contexts, leading to the intuition that the potential use of machine learning algorithms would support the continuous monitoring of the patient’s psychophysiological state [29]. Continuous monitoring of motor activity, using WDs, has proven useful for detecting neurobehavioral alterations, such as delirium [30]. From this perspective, the adoption of wearables in healthcare pathways also appears to be a cost-effective choice not only because of the low initial investment, but also because it reduces hospitalization costs, optimizes staff deployment, and improves the efficiency of the healthcare system [31].

### 1.4. Limitations of Continuous Monitoring via WDs

The accuracy of monitoring via WDs can be affected by several factors: the lack of transparency in signal processing and handling, the sensitivity of sensors to environmental factors [32], sensor placement, patient population, and the physiological variables monitored. These factors highlight that some locations provide greater reliability than others [17].

Other factors that could limit adherence and encourage abandonment of the device, especially in complex clinical patients, are skin tolerability, failure to cover relevant clinical parameters, and physical, ergonomic, and management discomforts [33]. The optical sensors that are part of WDs, based on photoplethysmography (PPG) in relation to skin pigmentation, can overestimate oxygen saturation in subjects with dark phototypes, exposing them to a greater risk of undetected hypoxemia and possible clinical disparities [34,35]. Generating large amounts of data, WDs are often subject to signal loss, motion artifacts, and environmental interference. This variability requires careful selection of clinically relevant signals and automatic filtering strategies to avoid information overload, and redundant alerts, and reduce decision-making efforts for healthcare professionals, while ensuring the true clinical utility of the collected data [21]. A limitation of continuous monitoring via AI-integrated WDs is the low confidence of patients in the reliability and functioning of such technologies [36] and by healthcare professionals, where more scientifically validated information could reduce skepticism, encouraging the acceptance and use of these systems [37].

### 1.5. Digital Technologies: Medico-Legal and Ethical Implications

The use of WDs in postoperative monitoring, while identifying early signs of clinical deterioration and complications, raises concerns about accountability, security, and data transparency [23]. In case of adverse events, it is necessary to define precisely a chain of responsibility [24].

First, the lack of clear accountability between the software developer, the physician, or the institution using it is a major critical issue can lead to incorrect or biased decisions. Without shared accountability, trust in the system is undermined [3].

Second, the continued collection of biometric data, via WDs, raises questions about user consent, data protection frameworks, and the risks of misuse or breaches [38]. A governance model that includes ethical guidelines and periodic system audits is needed to ensure the trustworthiness of wearable AI systems [39]. Inequality of access, often determined by socioeconomic factors, privacy protections, and the existing regulatory vacuum, should not be overlooked, as current regulatory frameworks are often fragmented and incomplete [40]. Methods are needed that enable developers and policy makers to carry out a preventive ethical assessment aligned with the EU AI Regulation, to identify risks at the design stage [41].

Ultimately, responsible implementation is needed, including ethical guidelines, adequate training for healthcare professionals on the risks and proper use of digital health technologies, and interactive and accessible informed consent [42].

While technical capabilities of WDs are often emphasized, this narrative review does not aim to compare devices solely based on performance metrics or to establish a hierarchy of superiority. Instead, the central objective is to demonstrate that wearable devices are not interchangeable: each device is designed with specific sensor configurations, operational profiles, and validation contexts that make it more suitable for certain clinical scenarios than others. By analyzing nine clinically validated WDs, this review highlights the importance of context-driven selection, showing how matching the right device to the right setting can enhance clinical utility, patient safety, and workflow efficiency.

## 2. Materials and Methods

This review was conducted as a narrative synthesis of the current evidence on wearable multiparametric monitoring devices. The aim was not to perform a systematic comparison of technical specifications, but to critically analyze published and manufacturer-supplied data to explore how and in which clinical contexts each device has been tested, and to determine whether they are interchangeable or best suited to specific scenarios.

No financial, commercial, or personal interests related to the devices analyzed have been declared by the authors, and no conflicts of interest have influenced the selection, analysis, or interpretation of the data presented in this narrative review.

### 2.1. Literature Search Strategy

We conducted a narrative evidence synthesis following best practices for non-systematic reviews. Searches were run in MEDLINE/PubMed and Embase for records published from January 2019 through August 2025, in English or Italian. Clinical trial registries (ClinicalTrials.gov, EU CTR) and regulatory summaries (e.g., CE/FDA listings) were consulted to verify intended use and status.

Searches were conducted in multiple stages between May and August 2025, with August 2025 representing the final update and date of last access to registries and manufacturer documentation. This review followed recognized best practices for narrative syntheses, including the SANRA quality assessment framework and recommendations outlined by Ferrari and Greenhalgh [43,44,45].

Searches combined concept terms for continuous, non-invasive multiparametric monitoring (e.g., {wearable}, {biosensor}, {continuous monitoring}, {vital signs}, {remote monitoring}, {perioperative}, {ICU}, {emergency/prehospital}) with device-specific keywords (e.g., {Radius VSM^®^}, {BioButton^®^}, {Portrait Mobile^®^}, {VitalPatch^®^}, {CardioWatch 287-2^®^}, {C-Med Alpha^®^}, {SensiumVitals^®^}, {CheckPoint Cardio^®^}, {Isansys Lifetouch^®^}). Reference lists of eligible studies were hand-searched and forward citation tracking was performed to identify additional reports.

To complement peer-reviewed evidence with accurate technical specifications and integration features, we also consulted publicly available manufacturer documentation (instructions for use, brochures, technical and integration guides) for each device when relevant to clarify sensor configuration, connectivity, and intended use.

Eligibility focused on clinically oriented studies (validation, feasibility, comparative accuracy, or real-world deployment) involving multiparametric medical-grade wearables used in perioperative, post-operative, emergency, or post-ICU settings. Consumer-grade trackers without regulatory status were excluded. Where there was heterogeneity in designs and outcomes, findings were synthesized narratively using a standardized extraction template capturing device model, study design, clinical context, population, outcomes (accuracy, feasibility, workflow integration, acceptance), and funding source. Consistent with this scope, manufacturer technical reports were used to supplement device characteristics but not to replace clinical validation evidence; bench testing without human participants was generally excluded (with clearly labeled exceptions where operational feasibility in high-motion settings informed context of use).

### 2.2. Devices Selection Criteria

Devices were selected through a preliminary screening of commercially available, clinically validated multiparametric wearable monitors. Inclusion required:

1. Regulatory clearance for medical use (e.g., CE marking, FDA clearance) in at least one major market.
2. Capability for continuous, non-invasive multiparameter monitoring.
3. Evidence of deployment in clinical settings, documented either in the peer-reviewed literature, observational studies, or manufacturer technical reports. To ensure relevance to current clinical practice, only devices with clinical validation published after March 2023 in perioperative, post-operative, or post-ICU settings were included. Devices lacking recent peer-reviewed evidence or clinical deployment in these contexts were excluded, even if previously certified.
4. Relevance to current clinical workflows, such as compatibility with hospital infrastructure or applicability in prehospital/emergency care.

In addition to the predefined requirements, further exclusion criteria were applied:

1. A lack of acceptable accuracy in at least three core vital signs.
2. Clinically unacceptable measurement bias compared with gold-standard references,
3. absence of pragmatic or outcome-oriented clinical validation beyond proof-of-concept studies.
4. Purely technical bench-testing without human subjects. One exception was made for C-Med Alpha°^®^, considered justified because, despite the limited body of peer-reviewed validation, the device has already been deployed in simulated emergency transport scenarios with documented feasibility. This positions it beyond mere bench-testing, providing preliminary evidence of translational relevance for operational settings.
5. Studies not specifying the device model or version.
6. Non-medical consumer-grade wearables without regulatory clearance for clinical use.

Based on these criteria, some devices were excluded, including Biobeat^®^ (regulatory 510(k) clearance indicates it is designed for spot-check use rather than continuous ward monitoring [46] and limited multiparametric coverage [47])—and ChroniSense Polso^®^, due to insufficient accuracy in RR, SpO_2_, and temperature measurements [48]. The nine devices retained in this review, Radius VSM^®^ (Masimo Corporation, Irvine, CA, USA), BioButton^®^ (BioIntelliSense Inc., Redwood City, CA, USA. Distributed by Medtronic), Portrait Mobile^®^ (GE HealthCare, Chicago, IL, USA), VitalPatch^®^ (VitalConnect Inc., San Jose, CA, USA), CardioWatch 287-2^®^ (Corsano Health B.V., The Hague, Netherlands. Distributed by Medtronic), Cosinuss° C-Med Alpha^®^ (Cosinuss Gmb, Munich, Germany), SensiumVitals^®^ (Sensium Healthcare Limited, Abingdon, Oxfordshire, UK), Isansys Lifetouch^®^ (Isansys Lifecare Ltd., Abingdon, Oxfordshire, UK), and CheckPoint Cardio^®^ (CheckPoint R&D LTD., Kazanlak, Bulgaria), fulfilled all inclusion criteria and demonstrated acceptable accuracy and clinical applicability in the specified settings. Based on the selection criteria, certain devices were excluded (for example, Biobeat Platform-2^®^ and ChroniSense Polso^®^, as stated in Limitations and Considerations).

The final selection represents a cross-section of technologies currently available for use in general wards, perioperative care, remote monitoring, and emergency settings.

### 2.3. Data Extraction and Synthesis

For each included source, we recorded:

- Device name and version;
- Study design (randomized trial, observational, pilot, manufacturer report);
- Clinical context (e.g., general ward, ICU, emergency department (ED), prehospital, remote/home monitoring, austere environment);
- Population and sample size;
- Key outcomes (accuracy, feasibility, integration into clinical workflow, patient acceptance);
- Funding source (independent vs. manufacturer-supported).

## 3. Cross-Device Analysis

The nine devices analyzed in this review meet basic requirements for modern medical-grade wearables: wireless communication, lightweight design, multiparametric capability, and certification for clinical use (CE and/or FDA).

To provide a clear comparative view, Table 1 summarizes the core specifications of each device (such as monitored parameters, measurement technology, form factor, and battery life) together with the clinical settings in which they have been tested. Highlighting these features side by side allows for an immediate appreciation of where the devices diverge, both technologically and operationally, setting the stage for the subsequent discussion on why their use should be context-specific rather than interchangeable.

## 4. Interoperability and Integration Challenges in Clinical Practice

The successful implementation of wearable devices in postoperative monitoring hinges not only on their technical performance but also on their ability to integrate seamlessly into existing clinical infrastructures. Interoperability, defined as the capacity of devices and systems to exchange, interpret, and use data consistently, is a critical enabler of this integration.

The nine devices differ not only in sensor configuration and monitored parameters, but also in their wireless communication protocols, a factor that can significantly affect clinical implementation and interoperability.

While most employ Bluetooth Low Energy (BLE) to connect with a gateway or mobile device, some integrate hospital-grade Wi-Fi (e.g., Radius VSM^®^, Portrait Mobile^®^, Isansys Lifetouch^®^) or specialized Medical Body Area Network (MBAN) protocols.

For example, Radius VSM^®^, BioButton^®^ combines BLE with cloud-linked Wi-Fi for remote patient monitoring, optimized for low-power, long-duration use. Portrait Mobile^®^ uses MBAN for dedicated bandwidth in hospital wards, while devices such as C-Med Alpha^®^ and VitalPatch^®^ rely on BLE-to-gateway communication, which can be advantageous in mobile or austere settings without permanent Wi-Fi coverage.

These differences are not trivial and highlight important observations:

1. Infrastructure dependency: Devices requiring secure in-hospital Wi-Fi or MBANs may achieve lower latency and higher reliability in real-time streaming, but their deployment is limited to facilities with compatible infrastructure.
2. Interoperability: Use of open, standards-based protocols (BLE, Wi-Fi) can facilitate integration with third-party clinical platforms and Electronic Health Record (EHR) systems, while proprietary or gateway-restricted protocols may lock the device to a vendor ecosystem, complicating multi-vendor environments.
3. Security and compliance: Protocol choice also determines the encryption standards and authentication mechanisms available, which are critical for compliance with healthcare data protection regulations (e.g., GDPR, HIPAA).

Among the devices analyzed, Portrait Mobile^®^, Isansys Lifetouch^®^ explicitly declares compatibility with IHE/HL7 standard (Health Level Seven International, Ann Arbor, MI, USA) for ADT, trends and alarm messaging, facilitating integration into multi-vendor EHR systems. While public documentation does not explicitly confirm HL7 FHIR support for SensiumVitals^®^, middleware solutions and APIs could potentially be used to map HL7 v2 data into FHIR-compatible formats, aligning with modern interoperable frameworks. The system has also been successfully integrated into customized mHealth applications, demonstrating its adaptability and potential for enhancing clinical workflows through structured data exchange and visualization.

The Checkpoint Cardio^®^ supports HL7 FHIR through its mobile gateway, enabling structured and interoperable data exchange with hospital systems. Its API framework includes RESTful endpoints and configurable CSV exports, allowing for seamless integration with EHRs, HIS platforms, and third-party clinical applications. The gateway acts as a middleware layer, aggregating sensor data and translating it into standardized formats suitable for real-time monitoring and predictive analytics.

C-Med Alpha^®^ supports HL7 FHIR via its gateway and API architecture, enabling structured data exchange. The cosinuss° Health Web platform offers REST/FHIR interfaces and configurable CSV exports, allowing for integration with HIS/EHR systems. While BLE ensures compatibility with mobile health platforms and IoT gateways, direct integration with hospital EHR systems typically requires intermediary gateways capable of aggregating and translating BLE data into clinical formats. In this context, the mobile gateway enables structured data exchange via REST APIs and HL7 FHIR standards, supporting scalable and interoperable remote monitoring workflows.

CardioWatch 287-2^®^, while not universally documented as FHIR-native, has been effectively deployed in a fully FHIR-based EHR ecosystem and provides REST API access for integration. It offers SDKs for iOS/Android and secure HIPAA/AVG-compliant cloud, and integration with EHRs can be facilitated through middleware engines, which enable translation of data into standard formats such as HL7 and HL7 FHIR.

Although explicit support for HL7 FHIR is not consistently documented for BioButton^®^ and VitalPatch^®^, the use of middleware solutions such as Rhapsody, or via proprietary platforms or APIs as for Radius VSM^®^, suggests likely compatibility with interoperable standards, subject to customization based on the hospital’s IT infrastructure. Finally, its cloud-based architecture ensures scalability and HIPAA compliance, providing secure and reliable handling of patient data.

The platform of Isansys Lifetouch^®^ is built around the Patient Status Engine (PSE), which supports HL7 v2 messaging and RESTful APIs for integration with Electronic Health Records (EHRs) and clinical dashboards. The API architecture allows for real-time access to structured data streams, enabling automated calculation of early warning scores (EWSs) and integration into clinical decision-support systems. However, the system’s reliance on gateway infrastructure and default alert thresholds may require customization to avoid false positives in high-motion or low-perfusion scenarios.

### Comparative Limitations and Strengths

The nine wearable devices analyzed in this review exhibit distinct technical profiles that influence their clinical applicability. Below is a comparative synthesis of their key differentiating features:

- Completeness of Parameters: Radius VSM^®^ and VitalPatch^®^ offer broader multiparametric data, including optional EtCO_2_ and fall/posture detection, respectively. CardioWatch 287-2^®^ and CPC12S^®^ provide ECG and cuffless BP via PPG and dry electrodes, enhancing cardiovascular assessment. C-Med Alpha^®^ focuses on core vitals (HR, RR, SpO_2_, temperature) with high reliability in motion-intensive environments.
- Miniaturization and Comfort: BioButton^®^, SensiumVitals^®^, CardioWatch^®^, and Isansys Lifetouch^®^ stand out for patient comfort and usability in long-term wear. C-Med Alpha^®^’s in-ear design offers advantages in emergency and transport scenarios where chest access may be limited.
- Battery Life: BioButton^®^ leads in duration (up to 30 days), followed by VitalPatch^®^ (7 days) and SensiumVitals^®^ (5–7 days) depending on continuous monitoring requirements. C-Med Alpha^®^ is more suited to short-duration applications (<12 h).
- Connectivity and Integration: Portrait Mobile^®^, Radius VSM^®^, SensiumVitals^®^ and Isansys Lifetouch^®^ demonstrate strong hospital integration via Wi-Fi and compatibility with EMR systems. Portrait Mobile^®^ supports IHE/HL7 standards, while C-Med Alpha^®^, CardioWatch^®^ and CPC12S^®^ offer HL7 FHIR and REST API access. BioButton^®^ and VitalPatch^®^ rely on proprietary platforms, which may limit interoperability in multi-vendor environments.
- Data Security and Compliance: Devices using hospital-grade Wi-Fi or MBAN protocols (e.g., Radius VSM^®^, Portrait Mobile^®^, Isansys Lifetouch^®^ and SensiumVitals^®^) offer enhanced encryption and authentication mechanisms, supporting GDPR and HIPAA compliance. Open standards (BLE, FHIR) used by C-Med Alpha^®^, CardioWatch^®^, and CPC12S^®^ facilitate secure data exchange and scalability.

These distinctions underscore the importance of context-driven device selection. Rather than assuming functional equivalence, clinicians should consider sensor capabilities, integration potential, battery autonomy, and validation evidence to match each device to its optimal clinical setting.

## 5. Clinical Validation

The comparative analysis of the nine wearable devices demonstrates that, despite all being designed for continuous multiparametric monitoring, they differ substantially in accuracy, usability, and settings in which they have been validated. These differences translate into distinct strengths and limitations, suggesting that each device is better suited to specific clinical scenarios (Table 2) and patient populations, making them far from interchangeable.

To enhance clarity, the devices are presented in descending order according to the strength of available clinical evidence within each context, rather than by technical specifications or manufacturer claims. This structure allows readers to identify evidence gaps and contextual suitability at a glance.

### 5.1. Clinical Validation by Context of Use

(a) Perioperative/major surgery

Devices in this context must demonstrate high accuracy for HR, RR, and SpO_2_ under motion and variable perfusion, with seamless integration into perioperative monitoring systems. Strong validation in surgical wards or PACU is preferred.

1. SensiumVitals^®^ has been evaluated in several investigations in perioperative pathways, particularly in patients undergoing major surgery. Hernandez-Silveira et al. first demonstrated the feasibility of continuous patch-based monitoring in surgical wards, reporting acceptable accuracy for heart rate and respiratory rate [49]. In the TRaCINg study, the authors compared its measurements with nurse-recorded vital signs in postsurgical patients, confirming reliability and feasibility for early detection of deterioration [50,51]. Breteler et al. [52] conducted clinical validation studies in high-risk surgical populations, finding good agreement with reference standards for heart rate and respiratory rate, though temperature accuracy was less robust. Leenen et al. [53] further showed the practicality of implementation in postsurgical wards, emphasizing workflow integration. Most recently, the multicenter stepped-wedge SHEPHERD trial [54] assessed clinical outcomes after postoperative implementation, representing the largest randomized evaluation to date. Collectively, these studies establish a growing evidence base for the perioperative use of SensiumVitals^®^ in surgical patients.

*Strengths: Multiple validation studies; largest RCT (SHEPHERD) in surgical patients; demonstrated reliability for HR and RR; feasible integration into surgical wards.*

*Limitations: Variable accuracy for temperature; susceptibility to data loss or artifacts; clinical outcome data still heterogeneous.*

2. Radius VSM^®^ (Masimo) was assessed in the CONSTANT clinical validation trial on high-risk surgical patients, showing clinically acceptable agreement for HR (99.5%) and RR (96.3%) in procedures longer than 1.5 h [52].

Additional internal reports include the ongoing RECORD study (scheduled to conclude in December 2026) [55] conducted at the University Medical Center Groningen (Netherlands), which aimed to assess the reliability, accuracy, and applicability of data during perioperative and early postoperative periods.

Manufacturer data also highlight its modular capability, including the integration of EtCO_2_ monitoring for advanced perioperative use.

*Strengths: High accuracy under challenging conditions; integration with SafetyNet™ platform for perioperative monitoring.*

*Limitations: Broader clinical evidence remains limited; much of the data is manufacturer-derived.*

3. Isansys Lifetouch^®^ has been tested in perioperative settings. One clinical study described its feasibility in post-operative surveillance following laparoscopic bariatric surgery, reporting continuous monitoring with the device as practical and acceptable [56]. A randomized clinical trial [57] evaluated the use of Isansys Lifetouch^®^ for continuous vital sign monitoring in patients undergoing major noncardiac surgery, demonstrating improved postoperative outcomes and feasibility of integration into surgical ward workflows. However, large randomized controlled trials specifically in ICUs remain limited.

*Strengths: Demonstrated feasibility in post-operative surveillance; integrated into hospital workflows including National Early Warning Score (NEWS) calculation; supports continuous multiparametric monitoring with ECG-derived RR and optional SpO_2_; validated in surgical wards and pediatric populations.*

*Limitations: Limited accuracy under motion and poor perfusion; high rate of false positives when used for automatic NEWS2 scoring; only partial data completeness in some studies; lack of large-scale randomized trials in ICU settings.*

4. VitalPatch^®^ (VitalConnect) has been tested in perioperative and postoperative surgical ward contexts, with acceptable accuracy for HR, SpO_2_, and temperature in controlled postoperative care unit (PACU) settings had reported moderate-to-strong correlations for heart rate and acceptable agreement for SpO_2_ and temperature compared with standard monitors, although RR correlation remained low [58]. Crucially, these results come from static, controlled environments, with patients recovering in bed post-surgery. This context supports the feasibility of VitalPatch^®^ for stable inpatient monitoring.

*Strengths: Multiparametric coverage with additional posture/fall detection.*

*Limitations: Respiratory rate accuracy lower in dynamic conditions; limited validation in mobile/ambulatory perioperative patients*.

5. CheckPoint Cardio^®^ was evaluated in the NIGHTINGALE validation study, which included high-risk surgical patients in perioperative and high-intensity hospital settings. Results demonstrated high accuracy for heart rate and acceptable performance for RR and SpO_2_, with some limitations under motion and poor perfusion. These findings support potential application in perioperative surveillance, though further outcome-oriented studies are needed [59].

*Strengths: Suitable for extended surveillance in perioperative, post-operative, and general wards.*

*Limitations: Performance may degrade under motion or low perfusion; short battery autonomy (~24–48 h); limited evidence in emergency and home-care contexts; requires continuous connectivity and proper gateway setup for optimal alerting.*

6. CardioWatch 287-2^®^ (Corsano) has been applied in acute cardiovascular care and arrhythmia detection; it demonstrated reliable HRV and AF detection in static perioperative contexts. In controlled settings such as cardiac catheterization labs, it has shown promising accuracy [60,61]. In prospective studies, good agreement was demonstrated for RR intervals and HRV compared to standard ECG [62]. However, its performance in dynamic or ambulatory environments remains to be fully validated. These findings, from peer-reviewed validation studies conducted in controlled hospital environments, support its potential use in stable inpatient scenarios, while further research is warranted to assess its applicability in more variable clinical contexts, including post-operative care.

*Strengths: Comfortable wrist-worn form; cuffless BP measurement.*

*Limitations: Limited validation in high-motion emergency settings.*

(b) Post-ICU/general ward

This setting requires devices with extended battery life. In digitally mature hospitals, interoperability and compliance with HL7/FHIR standards are crucial features, enabling direct integration with EHR systems and clinical dashboards. Equally important are validated algorithms for early warning score generation, which help reduce alarm fatigue. Evidence derived from large-scale-ward cohorts is particularly valuable in this setting.

1. BioButton^®^ (BioIntelliSense) has been extensively tested in a large-scale retrospective observational study [1] involving 11,977 adult patients admitted to medical–surgical wards in multiple hospitals. The device was integrated with a real-time data platform capable of generating early alerts for clinical deterioration, with deployment in standard wards outside critical care. This large-scale inpatient experience supports the BioButton^®^’s applicability for continuous surveillance of general ward patients and for integration into early warning and rapid response workflows.

*Strengths: Extended battery life (up to 30 days) ideal for continuous ward monitoring without frequent maintenance.*

*Limitations: Lacks continuous SpO_2_ streaming, limiting application in higher-acuity ward patients.*

2. SensiumVitals^®^ has been positioned by several studies as a solution for continuous monitoring in this context. Hernandez-Silveira et al. [49] and subsequent feasibility work showed that continuous patch-based monitoring can extend surveillance beyond ICU discharge. Iqbal et al. [18] reported on real-world ward deployment, describing clinical actions taken in response to SensiumVitals^®^ alerts.

Van Rossum et al. [63] analyzed the default alarm strategy (seven consecutive out-of-range measurements, ~14 min) and proposed adaptive approaches to balance sensitivity and alarm burden, directly addressing a key implementation challenge in the ward setting. The evidence thus supports SensiumVitals^®^ as a valuable adjunct to intermittent nursing observations during step-down care.

*Strengths: Well-studied in the general ward context; supports early detection after ICU discharge; real-world implementation data available; adaptive alarm strategies emerging.*

*Limitations: Alarm delays inherent in default algorithm; potential for alarm fatigue; data quality affected by motion artifacts; requires strong nursing workflows.*

3. The Isansys Lifetouch^®^ has been evaluated in several feasibility and pilot studies in step-down and general wards, demonstrating continuous recording of HR, ECG-derived RR, and optional SpO_2_ via integrated sensors [64]. Reports from NHS pilots and the NICE briefing describe integration into hospital workflows, including automatic calculation of NEWS scores and early warning alerts [65]. PSE feasibility of wireless monitoring and displaying alarm was also tested in 982 hospitalized children, clinically valid data for more than 50% of intended monitoring time [66]. The device was also tested in a postoperative general ward for early detection of serious adverse events (which occurred in 37% of patients, with 38% occurring during monitoring) [67].

However, in the COSMIC-19 study, its accuracy was limited, with only 59.7% of heart rate and 38.5% of respiratory rate measurements falling within acceptable ranges compared to standard methods [68]. Moreover, the use of Lifetouch data to generate NEWS2 scores resulted in a high rate of false positive alerts (over 85%), indicating that while the device supports continuous monitoring, it should not be directly integrated into existing clinical alert systems without adaptation.

*Strengths: detected serious adverse events in postoperative patients during real-world monitoring.*

*Limitations: limited measurement accuracy in COSMIC-19 studies, especially for RR.*

*Clinically valid data achieved in only ~50% of intended monitoring time in pediatric cohort. Requires careful calibration before integration into alert systems.*

4. VitalPatch^®^ was validated in low-resource wards for sepsis detection and demonstrated feasibility for continuous monitoring in surgical wards. Additional insight into the platform’s core sensing technology can be drawn from studies on its predecessor, the HealthPatch MD. Although hardware and firmware have since evolved, the fundamental sensing principles remain consistent. In a clinical trial evaluating multiple wearable devices, the HealthPatch MD showed good feasibility and overall accuracy for continuous multiparametric monitoring, except for respiratory rate (RR), which was limited by frequent artifacts. Episodes of tachycardia >180 bpm required manual ECG strip review, leading to the device being judged not fit-for-purpose in that form [69]. While these findings cannot be directly extrapolated to the current VitalPatch^®^, they do support the validity of the underlying sensing approach.

*Strengths: Added mobility benefits for recovering patients.*

*Limitations: RR accuracy lower in dynamic ward settings.*

5. For Radius VSM^®^, some real-world evidence come from institutional pilots, such as deployment in the Vanderbilt University ED [70] enabled by integration with the Masimo SafetyNet platform, a wireless continuous monitoring and tele-response system associated with improved patient outcomes in medical wards [71,72]. It was piloted in step-down and mobile ward units (e.g., Vanderbilt ED corridors and waiting areas), showing feasibility outside critical care.

*Strengths: Modular design allows for tailoring to ward patient needs.*

*Limitations: Integration currently tied to Masimo SafetyNet ecosystem.*

6. CheckPoint Cardio^®^ was evaluated in the NIGHTINGALE study in high-dependency units and surgical wards, providing continuous multiparameter monitoring. Published data confirmed acceptable agreement with reference monitors, suggesting its utility for extended surveillance in postoperative and general ward settings [59].

*Strengths: Supports continuous multiparametric monitoring in high-acuity environments.*

*Limitations: Performance may be affected by motion and poor perfusion. Limited published evidence outside perioperative and ward contexts. Battery autonomy restricts long-term use without recharging.*

7. C-Med Alpha^®^ (Cosinuss°) has been documented in postoperative monitoring outside intensive and post-anesthesia care units [73], partly driven by the absence of other approved systems, and in comparative studies with standard monitoring devices in non-cardiac surgery [74]. Evidence includes both peer-reviewed comparative studies and manufacturer field reports.

*Strengths: High reliability for temperature and SpO_2_ even in motion-intensive environments.*

*Limitations: Short battery life (<12 h) restricts extended perioperative surveillance.*

8. Portrait Mobile^®^ has shown excellent integration into electronic health records (EHRs) and early warning score systems, but its validation has been almost exclusively in hospital networks with secure Wi-Fi infrastructure, which limits extrapolation to other care environments. A study underscored challenges related to signal quality, alarm fatigue, and clinical responsiveness, particularly in the context of ward-based postsurgical care where it was revealed that patient mobility and environmental variability can affect device performance [75].

*Strengths: Optimized for use in secure Wi-Fi environments with dedicated bandwidth.*

*Designed for continuous monitoring in ward-based postoperative care.*

*Limitations: Not suitable for low-connectivity settings. Performance may be affected by patient mobility and environmental variability.*

9. CardioWatch 287-2^®^ feasibility in early signs of clinical deterioration was assessed in a pilot study conducted on 34 patients wearing the wristband on a general ward for 14 days. The quality of the recorded physiological data was sufficiently detailed for detecting sepsis-related changes [76].

*Strengths: Signal quality was sufficient to identify subtle physiological variations preceding overt clinical events.*

*Limitations: Lack of comparator or outcome linkage: The study assessed feasibility and data quality, but not clinical impact (*e.g.*, time-to-intervention or reduction in deterioration-related events).*

(c) Emergency and transport

Emergency and transport scenarios demand rapid application, robustness to motion/environmental stress, and short-term accuracy for triage decisions. Portability and autonomy outweigh long-duration monitoring.

1. In field applications, C-Med Alpha^®^ has been used in mountain rescue operations with the Bergrettung in Upper Austria and in air ambulance missions, including helicopter transport simulations and operational flights [73,77,78,79]. In these high-stress environments, the device contributed to triage and monitoring under extreme conditions, such as hypothermia risk and avalanche rescue, and supported decision-making for advanced interventions like ECMO transfer.

Taken together, these reports (many of which are operational case studies rather than large-scale trials) support the device’s suitability for prehospital and wilderness emergency care and suggest potential in short-term hospital monitoring and select home-monitoring contexts. However, evidence for its performance in extended surveillance or dynamic inpatient environments remains limited, underscoring the need for further prospective validation.

*Strengths: Rapid deployment, motion tolerance, in-ear design avoids chest-access issues in field care.*

*Limitations: Short operational duration limits extended transport monitoring.*

2. The VitalPatch^®^ has been deployed in high-acuity clinical environments such as trauma, sepsis, and major surgery, including use in resource-limited emergency departments, where it has demonstrated robust and reliable signal acquisition. Notably, it has been validated in multicenter trials in Rwanda for the early detection of sepsis and other deterioration events [80].

*Strengths: robust in resource-limited perioperative environments (*e.g.*, Rwanda ED).*

*Limitations: Respiratory rate accuracy lower in dynamic conditions* [58].

3. Radius VSM^®^ has been piloted in emergency department non-traditional zones, such as corridors and waiting areas at Vanderbilt, demonstrating mobility-friendly deployment for triage and continuous observation, and confirming feasibility outside critical care [70].

*Strengths: Modular sensors adaptable to varying acuity levels.*

*Limitations: Dependent on proprietary hospital Wi-Fi integration*.

4. SensiumVitals^®^ has limited but emerging evidence in emergency and transport contexts. Pilot studies [53] have demonstrated the feasibility of rapid application of the patch in emergency department observation areas, allowing for continuous monitoring while patients await definitive assessment. These reports highlight the potential role of the sensor in detecting early deterioration in crowded triage environments, where intermittent measurements may miss acute events. The device’s low-profile, wireless design also makes it technically suitable for pre-hospital transport, though formal published studies in ambulance or retrieval settings remain scarce.

*Strengths: Easy and rapid application; valuable for triage surveillance; technically suitable for pre-hospital care.*

*Limitations: Limited published clinical trials in ED/transport; lack of outcome data; uncertainty about integration into emergency workflows.*

(d) Home/remote monitoring

Remote surveillance prioritizes patient comfort, minimal maintenance, and multi-day to multi-week autonomy. Connectivity to cloud platforms and integration into chronic care workflows are critical.

1. BioButton^®^ has been widely used during COVID-19 for remote home-based surveillance and chronic care [81].

*Strengths: Long autonomy; patient comfort; cloud integration for clinician review.*

*Limitations: Non-continuous SpO_2_ monitoring.*

2. CardioWatch 287-2^®^ was used in the OAC-AFNET 9 study, a large-scale digital European case-finding project. The device identified atrial arrhythmias lasting >6 min in 5% of older adults (>65 years) without previously known atrial fibrillation or anticoagulation therapy, demonstrating feasibility for long-term arrhythmia detection in free-living conditions [82].

Manufacturer-supported data from the REMO-CARDIO study (press release) reported higher AF detection rates than 48 h Holter monitoring.

Additional validation includes blood pressure measurement meeting AAMI/ESH/ISO standards with strong correlation to invasive measurements (European Heart Journal—Digital Health) [83] and accuracy for SpO_2_, RR, and pulse rate in cardiac catheterization patients [61], demonstrating its high accuracy in static conditions.

*Strengths: HRV accuracy; integration potential with FHIR-compliant systems.*

*Limitations: Less suited for multiparametric acute monitoring at home.*

3. C-Med Alpha^®^’s versatility is further illustrated by the Telecovid study at Munich’s Klinikum rechts der Isar, which employed the in-ear sensor for remote home monitoring of vital signs in COVID-19 patients, generating real-time early warning scores via cloud-based analytics. Although results have not yet been published in the peer-reviewed literature, this deployment demonstrates the platform’s potential applicability beyond acute and prehospital care, extending to home-based surveillance for infectious diseases [84].

*Strengths: Accurate vital sign acquisition in non-hospital settings.*

*Limitations: Requires gateway; limited battery autonomy.*

4. SensiumVitals^®^ has mainly been studied in hospital settings but has also been conceptually evaluated for post-discharge monitoring. The platform supports connectivity via a cellular bridge, enabling transmission of continuous vital signs to hospital dashboards. Hernandez-Silveira et al. [49] originally described this remote capability. However, large-scale randomized data in the home setting remain lacking, and published experience is still confined to feasibility reports and economic analyses. Remote monitoring therefore represents an important but as yet underexplored frontier for SensiumVitals^®^ deployment.

*Strengths: Remote capability demonstrated; potential for reducing readmissions and length of stay; cost-effectiveness suggested in modeling studies.*

*Limitations: No large RCTs in the home setting; regulatory and organizational barriers; unclear responsibility for out-of-hospital alert response.*

### 5.2. Final Comparative Considerations

**Figure 2:**
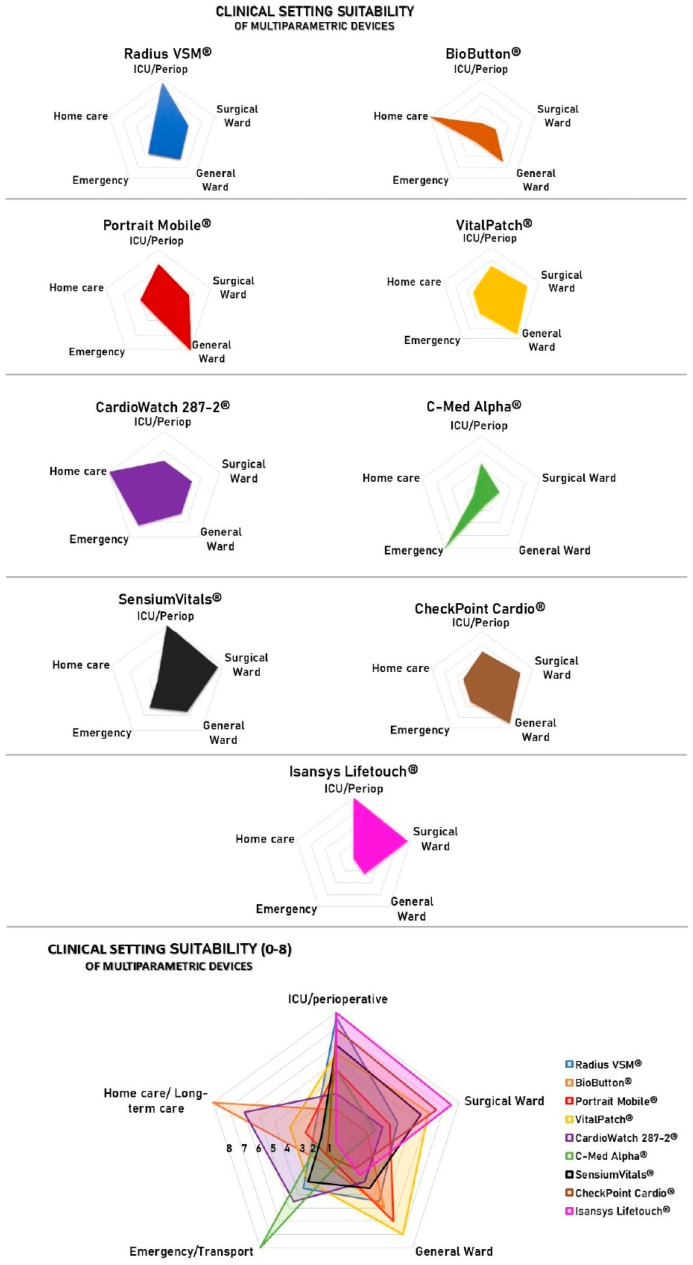
Comparative radar plots summarizing the key technical and clinical validation features of the wearable devices analyzed. The plotted profiles illustrate how device performance varies across domains, highlighting complementary strengths and limitations that support a context-driven rather than interchangeable use. The score, from 1 to 8, reflects a composite qualitative score integrating (I) extent of clinical validation (peer-reviewed RCTs, cohorts, manufacturer reports), (II) robustness of technical performance (accuracy, interoperability, autonomy), and (III) appropriateness for the target clinical context. Scores of 0–3 indicate preliminary evidence, 4–6 moderate validation, and 7–8 robust validation across multicenter or real-world deployments.

In synthesizing the available evidence, a clear pattern emerges linking device performance and design features to specific clinical environments (Figure 2).

In perioperative and high-acuity inpatient settings, devices such as Radius VSM^®^ evaluated in prospective perioperative care showed clinically acceptable accuracy in vital signs monitoring (e.g., CONSTANT trial) [52]. VitalPatch^®^ [80] combined robust accuracy with proven integration into early warning and rapid response systems, and Isansys Lifetouch^®^ [56] had the strongest supporting evidence. In addition to this, SensiumVitals^®^ represents one of the most extensively studied patch-based monitors: in perioperative and surgical populations, multiple validation and feasibility studies ([49,50,52,53]) and the large stepped-wedge SHEPHERD trial [54] confirm feasibility and reliability for continuous monitoring. C-Med Alpha^®^ has also been used in postoperative monitoring outside intensive and post-anesthesia care units, with deployment demonstrating feasibility in stable inpatient surgical populations [73,74].

The Checkpoint Cardio^®^ is supported by robust perioperative and post-ICU/general ward validation from the NIGHTINGALE study, demonstrating clinically acceptable accuracy for multiple vital signs. Evidence for emergency/transport and home care is not yet available, indicating important knowledge gaps.

For prehospital, transport, and austere environments, compact and rapid-deployment solutions like the C-Med Alpha^®^ and, in selected cardiovascular emergencies, the CardioWatch 287-2^®^, offer advantages in portability and resilience to environmental stressors [62,78]. Radius VSM^®^ was recently piloted in Vanderbilt University’s ED in non-conventional monitoring zones (waiting rooms, corridors), demonstrating its wireless capability and modularity. However, results are only available via internal reporting and have yet to be peer-reviewed. In emergency and triage environments, SensiumVitals^®^ [53] highlights the feasibility of rapid application and continuous surveillance in ED observation areas, although robust outcome data are lacking.

In contrast, for prolonged low-intensity monitoring in general wards or home settings, devices with extended autonomy and patient comfort, such as the BioButton^®^ and CardioWatch 287-2^®^, appear most appropriate. BioButton^®^’s low maintenance requirements and long battery life have facilitated widespread use during the COVID-19 pandemic. Similarly, CardioWatch 287-2^®^ has been trialed in multi-week arrhythmia detection protocols and home-based monitoring of cardiac patients (AFNET [81], REMO-CARDIO).

Evidence for the PSE is strongest in post-ICU/general ward settings, supported by pilot studies and NICE evaluation [65]. Evidence for emergency/transport and home care is limited to feasibility reports, highlighting an area for future pragmatic trials.

C-Med Alpha^®^, though originally designed for post-operative monitoring, has also been used in home care settings for chronic disease management in post-COVID recovery [84].

Portrait Mobile^®^, while demonstrating strong workflow integration in networked hospital environments, remains limited by its reliance on secure in-hospital connectivity. In post-ICU and general ward care, real-world deployments [18], and alarm strategy analyses [63], SensiumVitals^®^ supports its role in early detection of deterioration, while also underscoring the need for adaptive alarm thresholds. Finally, SensiumVitals^®^ also supports home monitoring via a cellular bridge, with feasibility described in early reports [49], although large randomized studies in the domiciliary setting remain absent. Overall, the SensiumVitals^®^ evidence base is broader than for most other devices, but variability in data quality, temperature accuracy, and alarm management highlight the need for careful implementation. These distinctions reinforce that device selection should be guided by the interplay between clinical context, patient profile, and operational constraints, rather than assuming functional equivalence across platforms.

### 5.3. Future Perspectives and Research Directions

**Figure 3:**
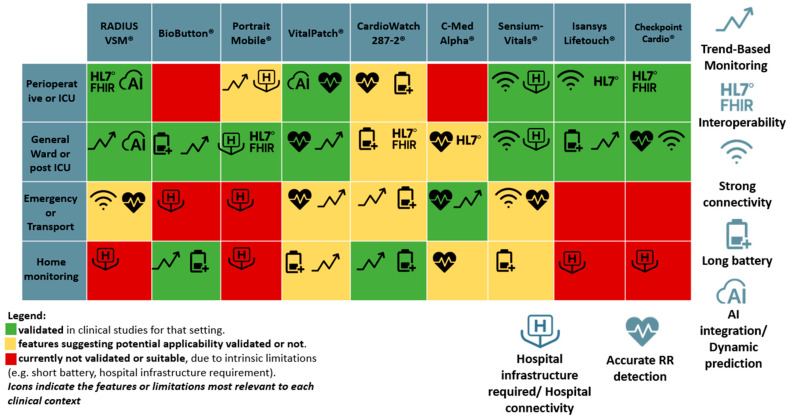
Color-coded matrix summarizing the suitability of each wearable device across different clinical contexts. Colors indicate the level of appropriateness based on available validation evidence and operational feasibility, while icons specify device-specific features (e.g., battery autonomy, interoperability, accurate RR detection) that support or limit its use in each setting.

Our comparative analysis confirms that wearable devices are not interchangeable but should be matched to specific clinical requirements and levels of validation (Figure 3).

Although current FDA-cleared devices already show feasibility across perioperative, ward, and remote settings, future innovation is especially promising in critical care. The ICU of tomorrow may evolve into a digital, interoperable ecosystem in which wearables communicate seamlessly with IoMT infrastructures, enabling continuous, multimodal monitoring across clinical units [85].

Recent advances in integrated sensing and wireless communication are progressively closing the gap between data acquisition and real-time transmission within IoMT infrastructures. For instance, Wi-Fi-based architectures enabling simultaneous sensing and data streaming, as demonstrated in capsule endoscopy systems [86], illustrate how similar frameworks could enhance connectivity and latency performance in wearable clinical monitoring.

Emerging paradigms such as IoMT-enabled health monitoring frameworks that integrate AI, cloud, and wireless networks [87], along with advances in smart textiles incorporating energy harvesting [88] (photovoltaic, piezoelectric, thermoelectric fabrics [89]), could extend monitoring beyond the capabilities of current wearables. Similarly, advanced signal processing techniques such as multi-scale entropy analysis of ECG signals can improve the accuracy and early detection of conditions like sleep apnea, demonstrating the potential of sophisticated algorithms combined with wearable sensors [90]. These innovations may help surmount persistent constraints like battery life, connectivity robustness, and data integration with hospital systems.

Projecting forward to 2050, anesthesiology and intensive care may adopt systems that support clinicians in higher-level decision-making rather than replacing their role [91]. In this vision, platforms such as ADAM (Awesome Data Acquisition Method) exemplify how real-time data aggregation from wearables, mobile apps, and clinical systems could underpin augmented monitoring and predictive analytics [92].

To transition from vision to practice, key research priorities include conducting multicenter randomized trials, evaluating clinical outcomes, performing cost-effectiveness studies, and deploying implementations in resource-limited environments. Only through this evidence-driven development can we bridge the gap between prototype or manufacturer-reported devices and sustainable, clinically trusted systems.

## 6. Limitations and Considerations

This review has several limitations. First, as a narrative rather than a systematic review, the study selection process may be subject to selection bias and does not guarantee full comprehensiveness of the available evidence. Based on the selection criteria, certain devices, despite being thoroughly evaluated, were excluded: Biobeat Platform-2^®^ was not included because clinically it was not intended as a continuous ward monitor, but as spot-check/intermittent measurement [46,47]; ChroniSense Polso^®^ was excluded due to insufficient accuracy for respiratory rate, SpO_2_, and temperature measurements [48].

Despite promising findings, the heterogeneity in data sources presents a challenge for direct comparison. Furthermore, only a few devices disclose full validation parameters (e.g., RMSE, Bland–Altman limits), which limits clinical decision-making in specific populations (e.g., low perfusion, movement artifacts, or comorbid patients). Still, the lack of published RMSE or bias limit objective comparison of measurement accuracy.

While most devices are CE- or FDA-certified (which may exclude other promising technologies currently under development or lacking regulatory approval), differences in sensor technology (PPG vs. ECG-derived RR), form factor (patch vs. wrist-worn vs. modular hub), and intended setting of use (ICU, ward, or home) suggest that no single device currently addresses all clinical needs. Finally, when selecting a device for clinical deployment, the choice of communication protocol should therefore be evaluated alongside measurement accuracy and usability, as it directly affects scalability, integration timelines, and the potential for interoperability across institutional and regional digital health networks.

Although the primary aim of this review was not to assess AI integration, it is noteworthy that some devices (for example BioButton^®^, already associated with AI-based platforms for trend analysis, or C-Med Alpha^®^, equipped with proprietary signal-quality algorithms) currently demonstrate advanced analytical capabilities, whereas others have the potential to incorporate predictive analytics in the near future to further enhance trend-based monitoring and early detection of clinical deterioration, as outlined in the Introduction. From this perspective, the accuracy and reliability of the collected physiological parameters will remain a fundamental prerequisite to ensure that predictive models can provide clinically meaningful and trustworthy insights.

Cost analysis was not conducted in this review, yet it remains a crucial factor for assessing the feasibility and large-scale integration of these devices into clinical workflows. Future research should prioritize head-to-head randomized trials and robust validation in diverse clinical populations.

In addition to cost considerations, the implementation of wearable devices entails substantial organizational challenges [24]. Clinical teams must dedicate time and resources to evaluate device options, integrate them into existing workflows, and provide staff training, all of which may delay or limit large-scale adoption. Furthermore, the rapid pace of technological change introduces additional barriers, ensuring compatibility between device components, maintaining reliable connectivity from sensors to hospital infrastructure and cloud platforms, and updating clinical pathways to align with evolving firmware and software ecosystems. These dynamic factors can significantly influence real-world feasibility and sustainability, even when technical accuracy and regulatory approval are assured.

## 7. Conclusions

Current evidence supports a context-driven selection of wearable monitoring systems. Rather than competing as interchangeable solutions, these devices provide distinct advantages when matched to the right clinical setting, patient profile, and operational requirements. Beyond technical performance and clinical validation, device selection must also consider the ethical and privacy implications previously outlined. In particular, the choice of a monitoring platform should account for its data governance model, compliance with relevant regulations, and transparency in handling sensitive patient information, factors that, like clinical accuracy, can influence suitability in different perioperative settings.

Future comparative trials should move beyond technical specifications and focus on patient-centered outcomes, cost-effectiveness, and integration into predictive care pathways.

## Figures and Tables

**Table 1:** Summary of the technical specifications and clinical validation contexts of the wearable devices included in this review. Acronyms used in the table are as follows: AI (Artificial Intelligence), AF (Atrial Fibrillation), API (Application Programming Interface), BLE (Bluetooth Low Energy), BP (Blood Pressure), ECG (Electrocardiogram), EHR (Electronic Health Record), EMS (Emergency Medical Service), EtCO_2_ (End-tidal Carbon Dioxide), FHIR (Fast Healthcare Interoperability Resources), GDPR (General Data Protection Regulation), HIS (Hospital Information System), HL7 (Health Level Seven), HR (Heart Rate), HRV (Heart Rate Variability), ICU (Intensive Care Unit), IoT (Internet of Things), ISO (International Organization for Standardization), MBAN (Medical Body Area Network), OR (Operating Room), PACU (Post-Anesthesia Care Unit), PPG (Photoplethysmography), RCT (Randomized Controlled Trial), REST (Representational State Transfer), RR (Respiratory Rate), SOFA (Sequential Organ Failure Assessment), SpO_2_ (Peripheral Oxygen Saturation), Wi-Fi (Wireless Fidelity), and WD (Wearable Device).

Device	Device Type	Measured Parameters	Wireless Technology	Interoperability	Interface and Dimensions	Battery Life and Charging	Data Display Interface	Certifications Countries of Use and Manufacturing Origin	Measurement Frequency	Ingress Protection	Strengths
**RADIUS VSM^®^** **(Masimo Corporation, Irvine, CA, USA)**	Modular wearable sensor Composed of replaceable components (3 electrodes, Masimo SET) Compatible with Masimo NIBP system (arm cuff for non-invasive blood pressure monitoring)	ECG (I, II, III, aVR, aVL, aVF), HR, RR, SpO_2_, temperature, posture, falls, arrhythmia detection, NIBP	Bluetooth Low Energy, integrated Wi-Fi (SafetyNet)	Proprietary API; FHIR via customization.	122 g + 22 g replaceable parts; charger: 22.9 × 9.4 × 5.4 cm; 203 g	12 h (up to 96 h in newer models), replaceable module	Tablet, smartphone (iOS/Android), hospital cloud (HCP)	FDA 510(k), CE NL Netherland (RECORD study) USA (Vanderbilt University)	SpO_2_, PR, RR every 30 s (0.033 Hz)	IP67	High accuracy in critical conditions, flexible system
**BIOBUTTON^®^** **(BioIntelliSense Inc., Redwood City, California, US**. Distributed by Medtronic)	Adhesive circular patch	HR, RR, temperature, SpO_2_	Bluetooth + Wi-Fi (cloud storage)	FHIR not explicit; middleware/API likely.	Ø 3.5 cm, 1 mm thick, ≤10 g	30 days (min. 14), disposable	Smartphone (iOS/Android), online dashboard (HCP)	FDA 510(k), CE USA (retrospective study)	HR, RR, Temp every 60 s	IP67	Long duration, ideal for hospital-to-home transition
**PORTRAIT** **MOBILE^®^** **(GE HealthCare, Chicago, IL, USA)**	Multiparametric module	SpO_2_, PR, RR	Wi-Fi MBAN	HL7/IHE supported; FHIR via middleware.	Handheld unit + cables, 223 g, 3.7″ display	3–5 days, USB base	GE workstation, hospital tablet (HCP)	FDA, CE, iF Design Award USA (Hospital environment)	SpO_2_, PR, RR every 10 s (0.1 Hz)	Some modules IP67	Designed for mobile patients, wireless monitoring
**C-MED°** **ALPHA^®^** **(Cosinuss Gmb, Munich, Germany)**	Ear-worn multiparametric sensor	Core temperature, HR, SpO_2_, perfusion	Bluetooth BLE (to gateway)	Native HL7 FHIR via gateway/API. cosinuss° Health Web REST/FHIR; BLE via gateway for EHR.	58.6 × 55.2 × 10.0 mm, 6.5 g; adaptable to ear (3 sizes)	15–48 h (continuous or intermittent), magnetic USB dock	Smartphone (iOS/Android), HCP	CE Class IIa DE Germany (Telecovid study) AT Austria (Mountain Rescue)	PPG (HR, SpO_2_, PI) every 0.005 s (200 Hz); temp every 10 s; motion every 0.01 s (100 Hz)	IP67	Validated in harsh environments, prehospital emergencies
**VITALPATCH^®^** **(VitalConnect Inc., San Jose, CA, USA)**	Adhesive patch	ECG, HR, RR, temperature, posture, falls	Bluetooth BLE (to gateway)	FHIR not explicit; middleware/API likely. Secure Cloud.	95 × 61 × 7 mm, ~13 g	7 days, disposable	Tablet, cloud, EMR integration	FDA 510(k), CE USA (PACU, trauma, surgery) RW Rwanda (emergency department) CH.	ECG every 0.008 s (125 Hz); HR, RR every 4 s (0.25 Hz)	IP67	Advanced integration with clinical systems
**SENSIUM** **VITALS^®^** **(Sensium Healthcare Limited, Abingdon, Oxfordshire, UK)**	Wireless adhesive patch chest sensor	HR, RR, Skin temperature, activity, posture	Proprietary RF (868 MHz EU), connected to Sensium Bridge	HL7/IHE; FHIR possible via API/middleware	14 g	Up to 5 days	Mobile app, desktop dashboard, nurse station	FDA 510 (k), CE GB United Kingdom (West Middlesex University Hospital, London) NL; FR; USA Netherlands and France Hospital system	Every 2 min	IP54 (shower proof)	Early detection of deterioration (e.g., sepsis, AF), seamless roaming, patient comfort
**CARDIOWATCH 287-2^®^** **(Corsano Health B.V., The Hague, Netherlands**. Distributed by Medtronic)	Multiparametric wearable sensor, wristwatch-like	ECG (single-lead), HR, HRV, RR, temperature, SpO_2_, AF detection, cuffless BP, GSR, motion	Bluetooth BLE (to gateway)	REST API; FHIR via middleware.	42 × 25 × 10 mm, 19 g	Up to 11 days (intermittent use)	Smartphone, tablet, HCP dashboard	FDA 510(k), CE EU Europe (OAC-AFNET 9 study) NL Netherlands (cardiac catheterization studies)	PPG (HR, SpO_2_, PI) every 28 s (32 Hz); ECG spot every 0.007 s; RR every 28 s; BP every 30 min; Temp every 28 s	IP66 (not submersible)	Cuffless BP detection, integrated ECG (single-lead), ergonomic
**Isansys** **Lifetouch^®^** **(Isansys Lifecare Ltd., Abingdon, Oxfordshire, UK)**	single-use adhesive chest patch (ECG sensor (ECG → HR, ECG-derived respiration (EDR)); integrated with third-party pulse oximeters (Nonin WristOx) and BP devices.	HR (ECG), ECG-derived RR (EDR), SpO_2_ (NIBP optional)	Bluetooth BLE (to patient gateway→ central hospital server)	FHIR not explicit; middleware/API likely. HL7 v2	Small: 7.5 cm × 3.5 cm × 0.8 cm Medium: 8.5 cm × 4.0 cm × 0.8 cm	Up to 4/5 days continuously	Patient gateway (tablet Samsung with software Isansys) to Lifeguard Server: dashboard web-based	CE-marked (Class IIa); FDA 510(k), UKCA UK, DE, IN, NO, US, TR Tested in UK, Germany, India, Norway, USA (manufacturer documentation and CE/FDA listings)	HR and HRV 1000 Hz RR (derivate from ECG) every minute Activity and Posture: via 3-axis accelerometer, real-time Temperature: via Lifetemp sensor, measured every 10 s	IP22	High-resolution ECG sampling Integrated with early warning scoring systems (e.g., National Early Warning Score 2, NEWS2) Not intended for ICU or critical care patients Contraindicated in patients with pacemakers or neurostimulators
**Checkpoint Cardio^®^ (CheckPoint R&D LTD., Kazanlak, Bulgaria)**	“All-in-one” wearable multisensor Patch	HR, RR, SpO_2_, NIBP (cuffless/PWT estimations), ECG (2/3 or 12 Leads) body temperature, body position, and activity.	Bluetooth BLE (to patient gateway→central hospital server)	HL7 FHIR	Not publicly disclosed by manufacturer	24–48 h Manufacturer specifies a maximum application time of up to 5 days for electrodes	Patient gateway, Smartphone or tablet (app), dashboard web-based	CE-marked (Class IIa); IT, RO, UK, DE, NL, SE, BE (Tested in UK, Germany, Netherlands, Sweden, Belgium—source: NIGHTINGALE study protocol)	Not publicly disclosed by manufacturer	Not disclosed	High accuracy for HR (bias 0.0 bpm; 95% LoA: −3.5 to 3.4) Integrated into hospital workflows and early warning systems (NEWS2)

**Table 2:** Clinical contexts and most suitable wearable devices, with supporting validation evidence. The table summarizes the preferred devices for perioperative and high-acuity inpatient care, general wards, prehospital and emergency transport, long-term low-intensity monitoring, and highly networked hospital environments. Device selection is guided by the balance between validated accuracy, interoperability, patient comfort, and operational constraints.

Clinical Setting	Most Suitable Devices	Clinical Validation Studies	Rationale
**Perioperative and high-acuity inpatient care (OR, ICU, surgical wards)**	Radius VSM^®^ (Masimo), VitalPatch^®^ (Philips/VitalConnect) SensiumVitals^®^ (Sensium Healthcare) ^®^}, CheckPoint Cardio^®^ (CheckPoint Care), Isansys Lifetouch^®^ (Isansys Lifecare).	[49,50,51,52,53,54,55,56,57,58,59,60,61,62]	High accuracy in challenging conditions; validated in perioperative and high-acuity environments; integration with early warning and rapid response systems.
**General wards (continuous surveillance)**	BioButton^®^ (BioIntelliSense), VitalPatch^®^ (Philips/VitalConnect) CheckPoint Cardio^®^ (CheckPoint Care), Isansys Lifetouch^®^ (Isansys Lifecare).	[1,18,49,59,63,64,65,66,67,68,69,70,71,72,73,74,75,76].	Feasible in standard ward environments; capability for early detection of deterioration; acceptable accuracy for key parameters. Posture and fall detection makes VitalPatch^®^ suitable for geriatric and rehabilitation care.
**Prehospital and emergency transport (EMS, helicopter rescue, austere environments)**	C-Med Alpha^®^ (Cosinuss°), CardioWatch 287-2^®^ (Corsano Health)	[53,58,70,73,77,78,79,80]	Portable, rapid deployment, resilience to environmental stressors; tested in real-world rescue and dynamic cardiovascular care.
**Long-term low-intensity monitoring (home, step-down units, chronic disease management)**	BioButton^®^ (BioIntelliSense), CardioWatch 287-2^®^ (Corsano Health)	[49,61,81,82,83,84]	Extended battery life; patient comfort; proven feasibility in multi-week monitoring protocols.
**Highly networked hospital environments (with secure Wi-Fi/EHR integration)**	Portrait Mobile^®^ (GE HealthCare)	[75]	Seamless integration into EHR and early warning score systems; optimized for in-hospital workflows.

## Data Availability

No new data were created or analyzed in this study. Data sharing is not applicable to this article.

## Abbreviations

The following abbreviations are used in this manuscript:

AI	Artificial Intelligence
AF	Atrial Fibrillation
API	Application Programming Interface
BLE	Bluetooth Low Energy
BP	Blood Pressure
CIS	Clinical Information System
ECG	Electrocardiogram
ED	Emergency Department
EDR	ECG-Derived Respiration
EHR	Electronic Health Record
EMS	Emergency Medical Services
EtCO_2_	End-Tidal Carbon Dioxide
EWS	Early Warning Score
FDA	Food and Drug Administration
FHIR	Fast Healthcare Interoperability Resources
GDPR	General Data Protection Regulation
GSR	Galvanic Skin Response
EHR	Electronic Health Record
HIPAA	Health Insurance Portability and Accountability Act
HL7	Health Level 7
HR	Heart Rate
HRV	Heart Rate Variability
HIS	Hospital Information System
ICU	Intensive Care Unit
IHE	Integrating the Healthcare Enterprise
IP	Ingress Protection
LoA	Limits of Agreement
MBAN	Medical Body Area Network
NIBP	Non-Invasive Blood Pressure
NEWS	National Early Warning Score
PACU	Post-Anesthesia Care Unit
PI	Perfusion Index
PPG	Photoplethysmography
PR	Pulse Rate
RCT	Randomized Controlled Trial
RR	Respiratory Rate
RMSE	Root Mean Square Error
SOFA	Sequential Organ Failure Assessment
SpO_2_	Peripheral Oxygen Saturation
WDs	Wearable Devices
Wi-Fi	Wireless Fidelity
